# Effects of patient health literacy, patient engagement and a system-level health literacy attribute on patient-reported outcomes: a representative statewide survey

**DOI:** 10.1186/1472-6963-14-475

**Published:** 2014-10-07

**Authors:** Kimberly A Kaphingst, Nancy L Weaver, Ricardo J Wray, Melissa LR Brown, Trent Buskirk, Matthew W Kreuter

**Affiliations:** Health Communication Research Laboratory, Washington University, St. Louis, MO USA; Division of Public Health Sciences, Department of Surgery, Washington University School of Medicine, Campus Box 8100, 660 S. Euclid Ave, St. Louis, MO 63110 USA; Saint Louis University School of Public Health, St. Louis, MO USA

**Keywords:** Health literacy, Organizational attributes, Patient engagement, Health literate organizations

## Abstract

**Background:**

The effects of health literacy are thought to be based on interactions between patients’ skill levels and health care system demands. Little health literacy research has focused on attributes of health care organizations. We examined whether the attribute of individuals’ experiences with front desk staff, patient engagement through bringing questions to a doctor visit, and health literacy skills were related to two patient-reported outcomes.

**Methods:**

We administered a telephone survey with two sampling frames (i.e., household landline, cell phone numbers) to a randomly selected statewide sample of 3358 English-speaking adult residents of Missouri. We examined two patient-reported outcomes – whether or not respondents reported knowing more about their health and made better choices about their health following their last doctor visit. Multivariable logistic regression models were used to examine the independent contributions of predictor variables (i.e., front desk staff, bringing questions to a doctor visit, health literacy skills).

**Results:**

Controlling for self-reported health, having a personal doctor, time since last visit, number of chronic conditions, health insurance, and sociodemographic characteristics, respondents who had a good front desk experience were 2.65 times as likely (95% confidence interval [CI]: 2.13, 3.30) and those who brought questions were 1.73 times as likely (95% CI: 1.32, 2.27) to report knowing more about their health after seeing a doctor. In a second model, respondents who had a good front desk experience were 1.57 times as likely (95% CI: 1.26, 1.95) and those who brought questions were 1.66 times as likely (95% CI: 1.29, 2.14) to report making better choices about their health after seeing a doctor. Patients’ health literacy skills were not associated with either outcome.

**Conclusions:**

Results from this representative statewide survey may indicate that one attribute of a health care organization (i.e., having a respectful workforce) and patient engagement through question asking may be more important to patient knowledge and health behaviors than patients’ health literacy skills. Findings support focused research to examine the effects of organizational attributes on patient health outcomes and system-level interventions that might enhance patient health.

## Background

Research has demonstrated the critical relationships between patient health literacy and incidence of chronic disease, self-reported health, utilization of preventive health services, health knowledge, rates of hospitalization, and health care costs [1–5]. The effects of health literacy are thought to be based on *interactions* between patients’ skill levels and the demands of health care and social systems [3]. To date, patients’ skills have received more attention in health literacy research and practice than have attributes of health care organizations. However, a recent report from the Institute of Medicine Roundtable on Health Literacy [6] and federal policy initiatives [7, 8] have drawn new attention to the attributes of health literate health care organizations (i.e., organizations that make it easier for people to navigate, understand, and use information and services to take care of their health) [6]. Little research has compared the relative importance of patients’ health literacy skills and organizational attributes, such as having a respectful health care environment and quality provider-patient communication, in determining patient-reported outcomes of care.

Patients’ interactions with health care staff are important to patient outcomes [9, 10] and having a respectful workforce that avoids stigmatizing patients with limited health literacy is a key attribute of health literate health care organizations [6]. Ethnographic research has shown the important role receptionists play in quality and safety of repeat prescribing of medications in general practice [11], and an intervention study examined the effects of training office staff about health literacy on staff knowledge and intentions [12]. However, little is known about the importance of front desk staff as part of a health literate workforce.

The quality of provider-patient communication is another attribute of health literate health care organizations suggested to affect patient outcomes [6, 13–16]. In particular, patient engagement through question asking can enhance satisfaction and recall of information imparted during an office visit [17], and research has shown that interventions that improve patients’ question asking can increase adherence to treatment recommendations and other medical outcomes [15, 18–21]. The importance of patient engagement and question asking has also been emphasized in health literacy practice [22–24]. Previous research has shown that patient engagement and activation influence different health outcomes than individual health literacy skills [25, 26]. Despite this prior work, the relative importance of patient health literacy skills compared with health literacy-related attributes of organizations, such as having a respectful workforce, and patient engagement through question asking has not been investigated.

We examined this question using data from a representative statewide telephone survey of Missouri residents. We examined the extent to which respondents’ experiences with front desk staff, bringing questions to a doctor visit, or their health literacy skills were predictive of two patient-reported outcomes – whether or not respondents learned from a medical encounter and whether or not they made better choices about their health following that encounter. We hypothesized that respondents who had better experiences with front desk staff, who brought questions to their providers, and who had higher health literacy skills would report learning more from and making better choices following a doctor visit.

## Methods

### Sample

We administered a telephone survey to a statewide sample of Missouri residents using two sampling frames: household landline telephone numbers within area codes in Missouri and cell phone numbers covering the same area. Primary sampling units (PSUs) included the 115 counties within Missouri and secondary sampling units (SSUs) included landline and cell phone numbers within each county. We used a two-stage probability sampling design that first selected a sample of PSUs and then selected a sample of SSUs from each PSU. We used a screener to identify cell phone-only households, which tend to be younger, more transient, and from racial and ethnic minority groups [27]. One member of each sampled household was systematically selected to complete the telephone survey. We oversampled four counties previously identified as having a greater proportion of residents with limited health literacy [28]. Inclusion criteria included being age 18 or older, speaking English, and having a landline telephone or cell phone. Excluding disconnected numbers, business and fax numbers, the participation rate was 42%. Respondents received a $15 incentive for participation. This study was approved by the Washington University Institutional Review Board.

### Measures

The survey was developed based on a review of existing health literacy measures and related patient care constructs. We identified measures from multiple sources and adapted them for telephone administration in a general population sample [29–33]. The survey was reviewed during its development by a multi-institutional advisory board comprised of primary care physicians, health literacy practitioners, and health communication experts.

We assessed *patient-reported outcomes* related to their last doctor visit with two items: “After going to the doctor, do you feel like you know more about your health?” and “After going to the doctor, do you feel like you are making better choices about your health?” [yes, no, don’t know/not sure].

We assessed three possible *predictor variables* (i.e., front desk experience, patient engagement through question asking, patient health literacy). To assess front desk experience at their last doctor visit, we asked patients “Did the staff at the front desk make you feel comfortable?” [not at all, somewhat, very comfortable] and “Did the staff at the front desk treat you with respect?” [little or no, some, great respect]. We examined patient engagement through question asking at last doctor visit using the item: “Did you take a list of questions to ask the doctor?” [yes, no, don’t know/not sure]. Because this was a telephone survey, health literacy was assessed using one self-report item: “How confident are you filling out medical forms by yourself?” [not at all, somewhat, extremely confident; [34]]. This screener item has been validated against objective measures of health literacy including the S-TOFHLA [35] and REALM [36, 37], which require in-person administration.

We also assessed a number of sociodemographic and self-reported health covariates. Respondents answered items on self-reported health [38], having a personal doctor, time since last doctor visit, having any form of health care coverage, educational attainment, gender, race and ethnicity, age, and personal diagnosis with diabetes, asthma, high blood pressure, high cholesterol, depression, or cancer.

### Analysis

Sampling weights were generated by first computing base weights based on the fact that for each county, simple random samples of phone numbers were selected from each of two overlapping frames – landline and cell phone. Weights then accounted for nonresponse for each frame and were calibrated to marginal state totals for control variables (i.e., age, sex, race, education, county) that were hypothesized to be associated with key outcomes of interest. The final computed weights were applied to all analyses in order to reflect population distributions. The self-reported health of the sample was very similar to the Missouri population as described by the Missouri Behavioral Risk Factor Surveillance System [38], and the gender, race, and health insurance distributions for our sample did not differ significantly from that of Census data for the state [39].

Descriptive statistics were examined for all variables. Bivariate analyses using chi-squared tests were conducted to examine the associations between outcome (i.e., reporting knowing more about health and making better choices about health after seeing doctor) and predictor (i.e., front desk experience, bringing questions to visit, health literacy skills) variables. We then built multivariable logistic regression models to examine the independent contributions of the predictor variables. In these models, outcomes were dichotomized as yes vs. no/don’t know. Because of the high correlation between the two front desk experience variables, we created a summed score; “good front desk experience” was defined as those who felt very comfortable with front desk staff and felt that front desk staff treated them with great respect. We also tested pairwise interactions to examine whether patients’ health literacy skills modified the associations of front desk experience and bringing questions with the outcome variables. Covariates tested in the multivariable models were: number of diagnosed chronic diseases (continuous); self-reported health (very good/excellent vs. good/fair/poor); having a personal doctor (yes vs. no/don’t know); last time saw doctor (less than 12 months ago vs. other); having health care coverage (any vs. none); educational attainment (less than high school; high school degree/GED; some college/technical degree; college degree or higher); gender; race/ethnicity (White, Black, other categories); and age (continuous). Observations missing values for a variable were not included in analyses using that variable. Data were analyzed using SAS Version 9.3 (Cary, NC). Statistical significance was assessed as p < 0.05.

## Results

Most respondents to the statewide survey were White (85%), and had some form of health insurance (82%), a personal doctor (77%), seen a doctor within the last 12 months (86%) and some education beyond high school (58%). Half were women (52%) and the mean age was 47 years (Table 1).

**Table 1:** **Characteristics of statewide sample of adult Missouri residents (N = 3358)**

Variable	N	%
Health status		
Excellent	510	15.2
Very good	1143	34.1
Good	1061	31.6
Fair	464	13.9
Poor	175	5.2
Number of chronic diseases		
0	1304	38.9
1	919	27.4
2–3	994	29.6
4–6	138	4.1
Have personal doctor	2564	76.5
Last doctor visit less than 12 months ago	2871	85.5
Have private or public health insurance	2736	81.8
Female	1745	52.0
Educational attainment		
Less than high school	395	11.8
High school/GED	1005	30.0
Some college/Technical degree	1204	35.9
College degree or higher	748	22.3
Race/ethnicity		
White	2844	84.8
Black/African American	373	11.1
Hispanic/Latino	28	0.8
Asian American	39	0.2
American Indian/Alaska Native	47	1.4
Other	22	0.7
Health literacy^*^		
Not at all confident	172	5.3
Somewhat confident	1272	38.2
Extremely confident	1885	56.6
	**M**	**SD**
Age	47	17.9

*Health literacy was assessed using the screener item “How confident are you filling out medical forms by yourself?”^34^.

### Knowing more about health after seeing doctor

In bivariate analysis (Table 2), both of the front desk experience variables were significantly related to the outcome variable of respondents reporting that they knew more about their health after seeing a doctor (p < .0001), as was the combined front desk experience variable (p < .0001). In addition, respondents who brought questions to the visit (p < .0001) reported knowing more after the visit than those who did not. Respondents’ health literacy skills were not related to reporting knowing more after the visit.

**Table 2:** **Bivariate associations between patients’ front desk experience, question behavior, and health literacy skills, and patient-reported outcomes of care (N = 3358)**

	Patient-reported outcomes			
	Know more about health after seeing doctor		Make better choices about health after seeing doctor	
Variable	N (%)	p-value	N (%)	p-value
**Front desk experience**				
Comfort with front desk staff		<0.0001		<0.0001
Very comfortable	1963 (89.6%)		1858 (85.9%)	
Somewhat comfortable	742 (77.8%)		730 (78.4%)	
Not at all comfortable	55 (53.3%)		57 (56.2%)	
Treated with respect by front desk staff		<0.0001		<0.0001
Great respect	2035 (88.9%)		1912 (84.7%)	
Some respect	669 (77.4%)		671 (79.1%)	
Little or no respect	55 (57.6%)		61 (64.6%)	
Front desk experience		<0.0001		<0.0001
Good front desk experience	2266 (88.3%)		2143 (84.8%)	
Poor front desk experience	499 (72.2%)		506 (74.6%)	
**Patient question behaviors**				
Brought questions to visit		<0.0001		<0.0001
Yes	700 (90.1%)		671 (88.2%)	
No	2073 (83.3%)		1978 (80.6%)	
**Patient health literacy skills** ^*^		0.13		0.23
Extremely confident	1588 (86.1%)		1503 (82.2%)	
Somewhat confident	1045 (83.7%)		1016 (83.3%)	
Not at all confident	135 (82.5%)		124 (77.9%)	

*Health literacy was assessed using the screener item “How confident are you filling out medical forms by yourself?”^34^.

In a multivariable logistic regression model, having a good front desk experience and bringing questions were significant independent predictors of knowing more about health after a doctor visit (Table 3). Respondents who had a good front desk experience were 2.65 times as likely (95% confidence interval [CI]: 2.13, 3.30) to report knowing more about their health after seeing the doctor. Respondents who brought questions to the visit were 1.73 times as likely (95% CI: 1.32, 2.28) to report knowing more. Patient health literacy skill was not a significant predictor or moderator of this outcome.

**Table 3:** **Predictors of knowing more about health after seeing a doctor in a multivariable logistic regression model (N = 3202)**

Variable	Odds ratio	95% confidence interval
*Predictor variables*		
Good front desk experience	2.65	2.13, 3.30^**||**^
Brought questions to visit	1.73	1.32, 2.27^||^
Health literacy*		
Extremely confident	0.97	0.61, 1.55
Somewhat confident	0.91	0.57, 1.45
*Health-related covariates*		
Very good or excellent health^†^	1.49	1.19, 1.87^¶^
Have personal doctor	1.83	1.42, 2.37^||^
Having seen doctor in last 12 months	1.26	0.96, 1.65
Number of chronic diseases	1.01	0.92, 1.12
Having any medical coverage	1.00	0.76, 1.33
*Sociodemographic covariates*		
Educational attainment^‡^		
High school/GED	0.94	0.65, 1.36
Some college/Technical degree	0.71	0.49, 1.02
College degree or higher	0.69	0.46, 1.03
Female	1.11	0.90, 1.36
Age	1.00	0.99, 1.01
Race/ethnicity^§^		
Black	1.56	1.09, 2.22^#^
Other	1.88	1.04, 3.40^#^

*Referent category is not at all confident.

^†^Referent category is good, fair, or poor self-reported health.

^‡^Referent category is less than high school.

^§^Referent category is White.

^**||**^p < 0.001.

^¶^p < 0.01.

^#^p < 0.05.

In this model, self-reported health, having a personal doctor, and race/ethnicity were significant covariates. Respondents with very good or excellent health were more likely to report knowing more about health after a doctor visit than those with good, fair or poor health (odds ratio [OR]: 1.49; 95% CI: 1.19, 1.87), as were those with a personal doctor (OR: 1.83; 95% CI: 1.42, 2.37) compared to those without one. Compared with White respondents, those who identified as Black or African American (OR: 1.56; 95% CI: 1.09, 2.22) and those from another racial or ethnic group (OR: 1.88; 95% CI: 1.04, 3.40) were more likely to report knowing more about health after a doctor visit.

### Making better choices about health after seeing doctor

In bivariate analysis, both front desk experience variables were significantly related to the outcome variable of respondents reporting that they made better choices about their health after seeing a doctor (p < .0001), as was the combined front desk experience variable (p < .0001). Respondents who brought questions to the visit (p < .0001) also reported making better choices after the visit. Respondents’ health literacy skills were not related to reporting making better choices after the visit.

In a multivariable model, having a good front desk experience and bringing questions to the visit were significant independent predictors of making better choices after a doctor visit (Table 4). Respondents who had a good front desk experience were 1.57 times as likely (95% CI: 1.26, 1.95) to report making better choices about their health after seeing a doctor. Respondents who brought questions to the visit were 1.66 times as likely (95% CI: 1.29, 2.14) to report making better health choices after a visit. Patient health literacy skill was not a significant predictor or moderator of this outcome.

**Table 4:** **Predictors of making better choices about health after seeing a doctor in a multivariable logistic regression model (N = 3134)**

Variable	Odds ratio	95% confidence interval
*Predictor variables*		
Good front desk experience	1.57	1.26, 1.95^**||**^
Brought questions to visit	1.66	1.29, 2.14^**||**^
Health literacy*		
Extremely confident	1.07	0.68, 1.66
Somewhat confident	1.22	0.78, 1.90
*Health-related covariates*		
Very good or excellent health^†^	1.05	0.85, 1.30
Have personal doctor	1.94	1.52, 2.47^**||**^
Having seen doctor in last 12 months	1.57	1.22, 2.01^¶^
Number of chronic diseases	0.98	0.89, 1.08
Having any medical coverage	1.00	0.77, 1.31
*Sociodemographic covariates*		
Educational attainment^‡^		
High school/GED	0.83	0.57, 1.20
Some college/Technical degree	0.59	0.41, 0.86^¶^
College degree or higher	0.53	0.36, 0.79^¶^
Female	1.14	0.93, 1.38
Age	1.01	0.99, 1.01
Race/ethnicity^§^		
Black	2.70	1.81, 4.02^**||**^
Other	1.19	0.74, 1.92

*Referent category is not at all confident.

^†^Referent category is good, fair, or poor self-reported health.

^‡^Referent category is less than high school.

^§^Referent category is White.

^**||**^p < 0.001.

^¶^p < 0.01.

In this multivariable model, having a personal doctor, having seen a doctor within the last 12 months, educational attainment, and race/ethnicity were significant covariates. Respondents with a personal doctor were more likely (OR: 1.94; 95% CI: 1.52, 2.47) to report making better choices about their health after a visit than those without one. Those who had seen a doctor within the past 12 months were also more likely to report making better choices (OR: 1.57, 95% CI: 1.22, 2.01). Compared with those who had not completed high school, respondents with some college or a technical degree (OR: 0.59; 95% CI: 0.41, 0.86) and with at least a college degree (OR: 0.53; 95% CI: 0.36, 0.79) were less likely to report making better choices about their health after a doctor visit. Respondents who identified as Black or African American were more likely (OR: 2.70; 95% CI: 1.81, 4.02) to report making better health choices after a visit compared with White respondents.

## Discussion

The results of this representative statewide survey of Missouri adults may indicate that one attribute of a health care organization (i.e., having a respectful workforce) and patient engagement through question asking may be more important to patient knowledge and health behaviors than patients’ health literacy skills. We found that those who had a good front desk experience and brought questions to a doctor visit were more likely to report learning from and making better choices following the visit than their counterparts. In contrast, patients’ health literacy skills were not significantly related to either outcome, and this variable did not moderate the observed associations. The finding that at least one aspect of the health literacy demands of a health care setting may affect patients’ experiences more than their own skills suggests that creating a health literate health care organization that can respond effectively to the needs of all patients may be more critical than interventions to improve the health literacy skills of individual patients.

Front office staff have been described as the face of a health care setting, shaping patients’ first and last impressions of the organization [40]. These staff members have many important roles, including helping patients access care, complete medical and insurance forms, make appointments, and obtain prescriptions. Some attention has been given to the importance of training staff in communication skills [41, 42], although few studies have focused specifically on training staff in organizational attributes important to health literacy, such as the creation of a respectful and shame-free environment [6, 12, 23]. Our findings support the importance of respondents’ interactions with front desk staff as being significantly related to patient-reported outcomes of care. An important next step is to examine what types of interactions are driving responses to these variables; for example, whether responses are related to specific interactions with different types of front desk staff, frustration with the larger health care system, or a combination.

One mechanism by which interactions with front desk staff might impact patient outcomes is suggested by the finding that having a good front desk experience was a stronger predictor of reporting learning from a doctor visit than reporting making better choices after the visit. A possible explanation for this finding is that a person’s emotional response to a negative front desk experience might adversely affect their ability or motivation to process information in a doctor’s visit occurring only minutes later. Functional emotion theories posit that different emotions have different effects in generating, sustaining, and focusing a person’s cognitive activity [43]. Anger is generally evoked when obstacles interfere with goal-oriented behavior or one experiences demeaning offenses against oneself or one’s loved ones [44, 45], which could result from an unpleasant experience with front desk staff. This type of emotional reactance (i.e., unexpected, unintended anger) could interfere with or generate resistance to information from an affiliated source (e.g., a health care provider) [43, 46].

Our results also highlighted the importance of patient engagement in the medical encounter through bringing questions to the visit. While some low-intensity interventions to encourage patient question asking have not affected patient behaviors [22], other studies have shown that patient question asking can affect patient knowledge, satisfaction, adherence, and the quality of provider-patient interactions [19, 20, 47]. Because patients with limited health literacy have a complex array of communication challenges [48], which could impact their interactions with providers [22, 49–51], we tested for an interaction between bringing questions to the visit and patients’ health literacy skills. The lack of a significant interaction suggests that patient engagement through question asking is important for patients with varying levels of health literacy skills. This finding therefore supports a universal approach of encouraging all patients, not just those with limited health literacy skills, to bring questions to doctor visits [23, 24].

This study had a number of limitations. The telephone survey was cross-sectional, so we could not investigate direction of causality and prospective study designs are needed in order to examine these associations further. Respondents were asked to think about their last doctor visit in answering questions, and unpleasant or uncomfortable experiences may be more vivid in memories, leading to recall bias. Because the attributes of health literate health care organizations are a new area of inquiry, we did not find validated survey items in our literature review and we therefore adapted the items used here from existing related measures. The survey items were assessed for content validity by a transdisciplinary group of health care providers, researchers, and practitioners, but we did not further validate the items with a population-based sample. Therefore, although the patient-reported outcomes reflect patients’ experiences, they may differ from objective measures of improvement in comprehension or health behaviors following a doctor visit. Furthermore, the front desk experience variable captures only one aspect of the attributes of a health literate health care organization. Validated measures are greatly needed to advance inquiry regarding the effects of various attributes of health care organizations on health literacy outlined by the Institute of Medicine [6].

In addition, to limit participant burden and because of the time constraints of the telephone survey, we had only a limited number of questions for each construct. For example, we asked about front desk staff generally rather than specific roles, and it is not clear whether patients differentiated between different types of front desk staff. The identified associations between front desk experience and patient-reported outcomes should be investigated in greater depth in a future study. Finally, we used a self-report item to assess respondents’ health literacy skills and respondents may have overestimated their skill levels. Although this item has been validated against standard measures of health literacy skills [34, 52], it will be important to explore these results further with other measures of functional health literacy.

## Conclusions

Despite these limitations, the results of this representative statewide survey of Missouri adults highlights an important and novel area that has not generally been a focus of health literacy research, that at least some health literacy attributes of a health care organization and patient engagement may be more important to patient knowledge and health behaviors than the health literacy skills of individual patients. Much of the focus in health literacy research has been to examine the associations between individual-level patient skills and various health outcomes. These findings speak to the importance of expanding the focus of health literacy to examine the effects of attributes of health care organizations on patient outcomes and investigating how system-level interventions to improve health literacy organizational attributes and to encourage patient engagement can enhance patient health.
